# Comparison of Flavonoid Intake Assessment Methods Using USDA and Phenol Explorer Databases: Subcohort Diet, Cancer and Health-Next Generations—MAX Study

**DOI:** 10.3389/fnut.2022.873774

**Published:** 2022-04-04

**Authors:** Fabian Lanuza, Nicola P. Bondonno, Raul Zamora-Ros, Agnetha Linn Rostgaard-Hansen, Anne Tjønneland, Rikard Landberg, Jytte Halkjær, Cristina Andres-Lacueva

**Affiliations:** ^1^Biomarkers and Nutrimetabolomics Laboratory, Department of Nutrition, Food Sciences and Gastronomy, Food Innovation Network (XIA), Faculty of Pharmacy and Food Sciences, Institute of Nutrition and Food Safety (INSA-UB), University of Barcelona, Barcelona, Spain; ^2^CIBER of Frailty and Healthy Aging (CIBERFES), Instituto de Salud Carlos III, Madrid, Spain; ^3^Danish Cancer Society Research Center, Copenhagen, Denmark; ^4^Institute for Nutrition Research, School of Medical and Health Sciences, Edith Cowan University, Perth, WA, Australia; ^5^Unit of Nutrition and Cancer, Cancer Epidemiology Research Program, Catalan Institute of Oncology (ICO), Bellvitge Biomedical Research Institute (IDIBELL), Barcelona, Spain; ^6^Department of Public Health, Section of Environmental Health, Faculty of Health and Medical Sciences, University of Copenhagen, Copenhagen, Denmark; ^7^Division of Food and Nutrition Science, Department of Biology and Biological Engineering, Chalmers University of Technology, Gothenburg, Sweden

**Keywords:** food composition, polyphenol, aglycone, glycoside, reliability, concordance

## Abstract

Flavonoids are bioactive plant compounds that are widely present in the human diet. Estimating flavonoid intake with a high degree of certainty is challenging due to the inherent limitations of dietary questionnaires and food composition databases. This study aimed to evaluate the degree of reliability among flavonoid intakes estimated using four different approaches based on the two most comprehensive flavonoid databases, namely, United States Department of Agriculture (USDA) and Phenol Explorer (PE). In 678 individuals from the MAX study, a subcohort of the Diet, Cancer and Health-Next Generations cohort, dietary data were collected using three 24-h diet recalls over 1 year. Estimates of flavonoid intake were compared using flavonoid food content from PE as (1) aglycones (chromatography with hydrolysis), (2) aglycones transformed (converted from glycosides by chromatography without hydrolysis), (3) as they are in nature (glycosides, aglycones, and esters), and 4) using flavonoid content from USDA as aglycones (converted). Spearman's intra-class correlation (ICC) coefficient and weighted kappa (K) coefficient were calculated for the reliability analysis. When comparing PE total aglycones to USDA total aglycones, there was a moderate reliability when a continuous variable was used [ICC: 0.73, 95% confidence interval (CI): 0.70–0.76] and an excellent reliability when flavonoid intake was modeled as a categorical variable (K: 0.89, 95% CI: 0.88–0.90). The degree of reliability among all methods of estimated flavonoid intakes was very similar, especially between database pairs, for the flavanol subclass, while larger differences were observed for flavone, flavonol, and isoflavone subclasses. Our findings indicate that caution should be taken when comparing the results of the associations between flavonoid intakes and health outcomes from studies, when flavonoid intakes were estimated using different methods, particularly for some subclasses.

## Introduction

Flavonoids are the largest class of polyphenols, some of which are ubiquitous throughout the plant kingdom, while others are specific to one plant species/genus (1). Flavonoids are mainly found in plant-derived foods and beverages such as fruit, vegetables, nuts, cocoa products, tea, and wine (2, 3). In accordance with their chemical structure, flavonoids are often divided into six subclasses, namely, anthocyanidins, flavonols, flavanones, flavones, isoflavones, and flavanols or flavan-3-ols, which include monomers, proanthocyanidins, and flavanol-derived compounds (theaflavins and thearubigins) (2), although there are other existing subclasses (i.e., chalcones, dihydrochalcones, and dihydroflavonols) (4). In nature, they are usually found as glycosides, i.e., conjugated to a sugar. However, some flavonoids such as flavanols are only present as aglycones (free form) (1).

The estimation of dietary flavonoid intake is complex and challenging and is principally affected by the dietary assessment method and the food composition database (FCDB) used. Flavonoid composition data are influenced by the food (variety, origin, ripeness, terroir, etc.), processing and cooking method, and the laboratory methodology used (1).To date, there are two major global FCDBs for flavonoids, namely, the United States Department of Agriculture (USDA) and Phenol-Explorer (PE; www.phenol-explorer.eu) databases (5). The content of flavonoids in the USDA database is expressed as aglycones and is split into three separate databases, namely, flavonoids (512 foods/beverages) (6), isoflavones (560 foods/beverages) (7), and proanthocyanidins (285 foods/beverages) (8). The USDA data are reported as aglycones; using chromatography with/without hydrolysis, the USDA researchers converted the glycoside values into their aglycone forms. The PE database (release 3.6) includes data on all classes of polyphenols, including flavonoids, and reports the flavonoid content of 326 foods/beverages, measured by chromatography with hydrolysis (expressed as aglycones) and without hydrolysis (expressed as glycosides, aglycones, and esters) (9), and also includes retention factors for processing and cooking (10, 11).

Findings from several studies indicate that flavonoids may reduce the risk of chronic diseases, such as cardiovascular diseases, type 2 diabetes, and several types of cancers (12–15). Although the evidence is increasing, it is still not conclusive, and further research is warranted. Epidemiological findings regarding nutrition and health outcomes may differ due to the interaction between the exposome and the characteristics of the study population, as well as the study design, variable selection, and dietary assessment methods (16, 17). In particular, discrepancies in the results may be due to the different methodologies used to estimate flavonoid intake. This may be regarding the flavonoid subclasses included, the flavonoid FCDB used and its completeness, and the procedure/protocol utilized for the calculations between flavonoid FCDB (i.e., matching) and dietary assessment method (i.e., decomposition of complex foods) (18–20). Few studies have compared both databases on the estimation of flavonoids. Despite different approaches to the comparison methodology and statistical analysis applied, there is a consensus on the differences found between the estimates of flavonoid intake and subclasses by the database (19, 21–23). Mainly, it has been found that the USDA estimates were greater than the PE estimates for flavanol and anthocyanin subclasses, but the opposite was observed for flavonol, flavanone, and flavone subclasses. Furthermore, one of these studies showed highly correlated intake estimations for total flavonoids, flavanols, and flavanones (21). Also, some studies have compared flavonoid glycosides to aglycones, which could drive misinterpretation, and have not compared the three different methods (aglycones, aglycones transformed, and all forms) for estimating flavonoid intake from the PE database. To the best of our knowledge, there are no prior studies of reliability tests between databases on flavonoid intake estimations. The reliability tests used could be more sensitive to changes in consistency/absolute agreement than other isolated statistical tests (24). Thus, the aim of this study was to evaluate the degree of reliability among four methods of estimating flavonoid intakes using the USDA and PE databases.

## Methods

### Study Design and Subjects

This analysis is based on a validation subsample called the MAX study within the Diet, Cancer and Health-Next Generations (DCH-NG) cohort. The DCH-NG study, established in Denmark between August 2015 and April 2019, is an extension of the Diet, Cancer and Health (DCH) cohort (25). The DCH-NG cohort includes 39,554 participants with complete data collection and involves biological children (Generation 1), their spouses (Generation 1-Parent), and the grandchildren (Generation 2) of the participants in DCH (Generation 0) (26). From August 2017 until the end of January 2019, 720 participants of the MAX study, aged 18 or older, were enrolled, and both questionnaire data and biological samples were collected at baseline and at 6 and 12 months. All subjects completed two main questionnaires regarding lifestyle and dietary habits and participated in a health examination including the collection of biological samples and anthropometric and blood pressure measurements.

### Dietary Data

Besides completing the dietary questionnaire, participants in the MAX study also completed a 24-h dietary recall (24-HDR) at each time point using the web-based tool myfood24 (www.myfood24.org/) from Leeds University (27), which has been linked primarily with the Danish National Food Database and now contains ~1,600 Danish food items, including a recipe maker. A total of 676 participants had 1,436 complete 24-HDRs, and the data were calculated with the total intake of days for each analytical method by each individual. The participants reported all food consumed the day before the examination at the study center in grams by total portion size (as specified/selected by each participant). The portion sizes were based on reports from the Danish Food Institute. In complementary, the complex food products were calculated as recipes taking into account the individual ingredients and their corresponding proportions as estimated from standardized recipes, for example, the FFQ recipes from DCH cohort were used to standardize 24-HDR homemade recipes (28). Some recipes were mainly obtained from McCance and Widdowsons Food Composition Table versions 6 and 7 (29). For industrial packaged meals, the flavonoid estimation was done according to the percentages of ingredients in the food products. Over 450 types of food ingredients from a total of 6,000 food ingredients were used to estimate flavonoid intake, as previously described elsewhere (30).

### Assessment of Dietary Flavonoid Intakes

The USDA composition database contains flavonoid data in three separate databases: flavonoids (release 3.3), isoflavones (release 2.1), and proanthocyanidins (release 2.1). The flavonoid database includes five subclasses, namely, flavanols (monomers and flavanol-derived compounds), flavanones, flavones, flavonols, and anthocyanidins (6). Phenol Explorer includes the same flavonoid classes as USDA plus chalcones, dihydrochalcones, and dihydroflavonols (31). Four methods of dietary flavonoid intake estimation were developed for descriptive comparisons taking into account the database, analytical method, chemical structure, and subclasses in the following ways: (a) Phenol Explorer: (1) aglycone data provided with chromatography with/after hydrolysis or high-performance liquid chromatography for proanthocyanidins, (2) aglycones transformed from data provided from chromatographic analysis without hydrolysis where glycosides were determined and converted to aglycones using the molecular weight, and (3) total glycosides/all forms (expressed as they are in nature: glycosides, aglycones, and esters) provided with chromatography without hydrolysis; (b) USDA: (4) aglycones from total flavonoids (i.e., flavonoids, isoflavones, and proanthocyanidins). Additionally, a fifth method that considers only the flavonoid aglycone database (without isoflavones and proanthocyanidins) from the USDA was compared (Table 1). This method was excluded and presented in the supplementary information (Supplementary Table 1) because isoflavones and proanthocyanidins were missing. The USDA data were generated using chromatography with hydrolysis or chromatography without hydrolysis followed by the conversion of glycoside values into their aglycone forms using the molecular weight. Therefore, methods 2 and 4 are comparable, the only difference is the database used.

**Table 1:** Total flavonoids content by polyphenol databases and the methods of estimation used in MAX study.

	Flavonoid Databases				
	Phenol Explorer (PE)			USDA	
	**Total aglycones (mg/day)**	**Total aglycones transformed** **(mg/day)**	**Total glycosides^a^ (mg/day)**	**Total aglycones^b^** **(mg/day)**	**Aglycones (mg/day)**
Methods	Chromatography with/ after hydrolysis (1)	Chromatography without hydrolysis /Transformation (2)	Chromatography without hydrolysis (3)	Chromatography without hydrolysis / Transformation (4)	Chromatography without hydrolysis /Transformation (5)
Mean ± SD	378 ± 393	367 ± 392	427 ± 422	457 ± 608	197 ± 328
Median^c^ *(p25-p75)*	275 (116–524)	261 (106–511)	312 (140–592)	283 (122–592)	78 (36–187)
P20	90	80	110	93	30
P40	205	192	239	207	62
P60	361	348	415	370	105
P80	608	587	679	721	236

a *All forms (glycosides, aglycones, and esters), Phenol Explorer*.

b *Sum of flavonoids, isoflavones. and proanthocyanidins, USDA*.

c *Results are significant different by Wilcoxon (between all pairs) and Friedman tests (p < 0.001)*.

The overall procedure used to match the reported food items followed a stepwise protocol described by Knaze et al. (20). The first step was to convert the food items (food and recipes) from the 24-HDRs to ingredients. Second, we linked the ingredients from the 24-HDRs to the food items provided by the USDA and PE composition databases using an in-house software developed by the University of Barcelona, the Bellvitge Biomedical Research Institute (IDIBELL), and the *Centro de Investigation Biomédica en Red* (CIBER) (32). Third, the software calculated the intake of total, class, subclass, and individual flavonoids (mg/day) by multiplying the specific flavonoid content of the serving of each food item (expressed as mg/g food fresh weight) by the daily consumption of the selected food item (g/day).

### Statistical Analysis

Dietary flavonoid intake was presented as mean, median, and percentiles: 20^th^, 40^th^, 60^th^, and 80^th^. The contribution of each flavonoid subclass was estimated as the percentage of total flavonoids. Four methods were used in the food sources, flavonoid subclasses, and reliability analysis. The contribution of each food group to the flavonoid intake was calculated from each food item as a percentage. The nonparametric Wilcoxon and Friedman tests were used for comparisons. The reliability of the four flavonoid assessment methods was evaluated using: (i) intraclass coefficients (ICC) in which flavonoid intakes were assigned as continuous variables (mg/d); and (ii) kappa squared-weighted coefficients in which flavonoid intakes were assigned as categorical variables (quintiles). Additionally, Spearman's rank correlation coefficients (Spearman's rho) were calculated. The ICC was determined with the model of two-way mixed effect based on a confidence interval of 95%, which calculated the agreement in the content of flavonoids in each method under comparison: ICC < 0.5, 0.5–0.75, >0.75–0.90, and >0.90 are indicative of poor, moderate, good, and excellent reliability, respectively (24). The kappa coefficient was calculated as a measure of the agreement between flavonoid quintiles of each method. Kappa values < 0.4 are considered poor; 0.4–0.6 moderate; > 0.6–0.8 good; and > 0.8–1 excellent (33). All analyses were conducted using the SPSS Statistics software (version 27.0; IBM SPSS).

### Ethics

Danish Data Protection Agency and the regional ethical committees in Copenhagen and Aarhus (File (KF) 11–037/01) approved the Diet, Cancer and Health research project. The DCH-NG project was approved by the Danish Data Protection Agency (number 2013–41- 2043/2014–231-0094) and by the Committee on Health Research Ethics for the Capital Region of Denmark (number H-15001257). The study was conducted according to the guidelines in the Declaration of Helsinki.

## Results

The descriptive statistics are summarized by the method in Table 1. Median flavonoid intakes differed significantly across both databases and their applied methods. As expected, the estimation of total flavonoid intake was higher when using the method that quantified them as all forms as the chemical structure includes sugars. A slight difference between median PE total aglycones (275 mg/day), PE total aglycones transformed from glycoside data (261 mg/day) and USDA total aglycones (283 mg/day) was observed. Moreover, when comparing the estimated intakes of total aglycones from the PE and USDA databases, estimates were very similar until the 80^th^ percentile where they were higher using USDA values (721 mg/day) than PE values (608 mg/day).

The top dietary sources of flavonoids in the Danish population at investigation are presented by the method in Table 2. Cocoa products, fruit, and tea were the main contributors of flavonoids for all methods. The most commonly consumed cocoa products were chocolate, cocoa drinks, sauces, and cereal bars; and total aglycones coming from cocoa products were higher when estimated using PE (31.2 vs. 26.4% from USDA). For tea consumption, black and green tea infusions were the most frequently consumed, and total aglycones coming from tea were higher when estimated using the USDA databases (27.0 vs. 17.9% from PE). Estimates of total aglycones from fruit, whose main source of flavonoids was apples, were similar across all methods. The estimated percentage contribution from nuts and seeds, wine, and cereals and baked products was 1.7, 1.2, and 2.0 times higher, respectively, when estimated using PE compared to USDA. Conversely, the estimated contribution of vegetables to total aglycones was 1.2 times higher when estimated from USDA compared to PE. These differences in food sources are mainly due to the composition data on polyphenols (mg/100 g) applying the different methods, such as for cocoa products (Supplementary Table 2).

**Table 2:** Top food sources by databases and methods used in MAX study.

**Top food sources**	Flavonoid Databases			
	Phenol Explorer			**USDA**		
	**Total aglycones**	**Total aglycones transformed^a^**	**Total glycosides^b^**	**Total aglycones**
Food items (*n*)	955	912	912	1,030
Cocoa products (%)	31.2	33.8	29.1	26.4
Total fruits (%) - Apple (%)	20.7-12.0	20.5-10.7	21.1-11.9	19.5-10.8
Tea (%)	17.9	18.7	20.8	27.0
Nuts and seeds (%)	11.4	9.3	8.4	6.7
Wine (%)	6.1	6.5	6.6	5.3
Cereals and baked products (%)	5.4	5.6	7.4	2.7
Vegetables (%)	4.0	3.5	4.5	4.8
Cumulative percentage (%)^c^	96.7	97.9	97.9	92.4

a *Transformed (converted from glycosides by chromatography without hydrolysis)*.

b *All forms (glycosides, aglycones, and esters), Phenol Explorer*.

c *The residual percentage of food sources comes from oils, herbs, seasonings, and others beverages*.

**Figure 1:**
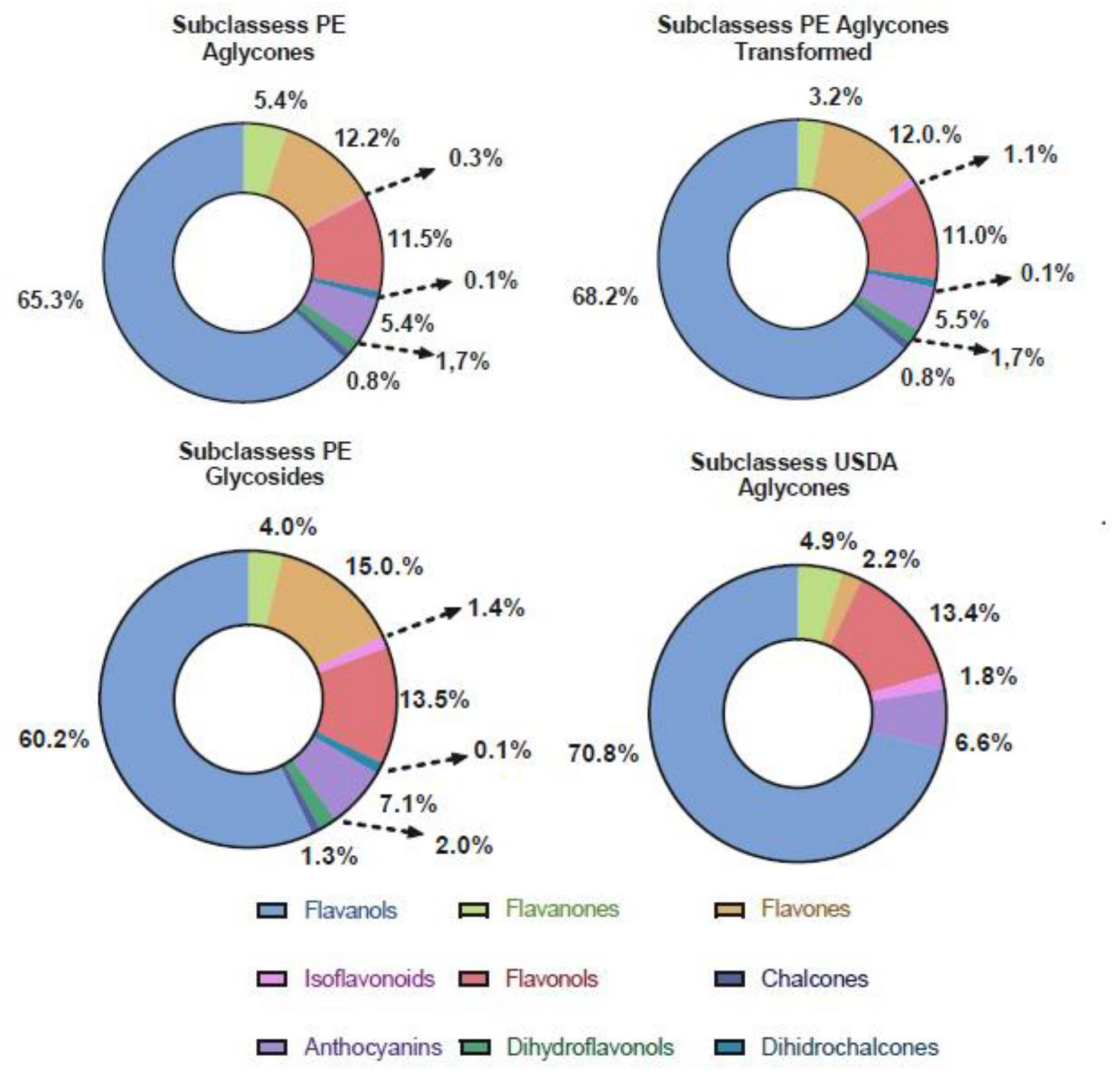
Total percentage distribution of flavonoid subclasses by databases and methods. The Phenol Explorer cake charts include chalcones, dihydrochalcones, and dihydroflavonols. Flavanols from USDA include the proanthocyanidin subclass.

The contribution of flavonoid subclasses by the method is expressed as median (p25-p75) in Table 3 and as percentages in Figure 1. Intakes of anthocyanins and isoflavones (aglycones) were higher when estimated from USDA than from PE, whereas the opposite was observed for flavones (Table 3). Estimated intakes of flavanols, flavanones, and flavonols were similar across both databases. When comparing the two PE aglycone methods, the estimated intakes of all subclasses were very similar, except for the flavanones, which were lower when using the transformed data (Table 3). Although flavonoid glycoside intakes are higher as net values for all subclasses when compared to both PE aglycone methods (Table 3), this is not reflected in the percentages of the total distribution (Figure 1).

**Table 3:** Total median of flavonoid subclasses by databases and methods.

**Subclasses**	**Methods**			
	**Phenol Explorer**			**USDA**
	**Total aglycones (mg/day)**	**Total aglycones transformed^a^** **(mg/day)**	**Total glycosides^b^ (mg/day)**	**Total aglycones** **(mg/day)**
Flavanols^c^	198.5 (62.5–431.4)	198.7 (62.5–432.1)	202.2 (63.2–444.6)	207.3 (71.3–504.2)
Flavanones	1.2 (0.3–5.2)	0.6 (0.1–2.4)	1.1 (0.1–4.5)	1.1 (0.2–10.0)
Flavones	10.5 (4.8–18.5)	8.7 (3.0–15.5)	17.5 (5.9–31.8)	1.0 (0.3–3.4)
Chalcones	0.1 (0.07–0.1)	0.1 (0.06–0.1)	0.1 (0.06–0.1)	-
Isoflavonoids	0.002 (0.00–0.01)	0.01 (0.00–0.1)	0.01 (0.00–0.1)	0.4 (0.1–0.9)
Anthocyanins	1.7 (0.3–21.6)	1.7 (0.3–20.7)	3.3 (0.5–33.6)	2.9 (0.5–27.8)
Flavonols	15.7 (5.9–34.6)	14.9 (4.6–33.6)	24.5 (6.8–57.9)	17.6 (9.0–29.8)
Dihydrochalcones	2.3 (0.4–3.5)	2.3 (0.4–3.5)	4.1 (0.8–6.0)	-
Dihydroflavonols	1.8 (0.5–11.1)	1.8 (0.5–11.1)	2.6 (0.8–16.3)	-

*Values are presented as median (p25-p75)*.

a *Transformed (converted from glycosides by chromatography without hydrolysis)*.

b *All forms (glycosides, aglycones, and esters), Phenol Explorer*.

c *Flavanols from USDA include the proanthocyanidin subclass*.

The degree of reliability and the correlation between flavonoid intake estimation methods are presented in Table 4. When comparing the two PE aglycone methods (total aglycones vs. total aglycones transformed), all reliability measures were excellent. When comparing PE and USDA aglycone methods, reliability was moderate for continuous flavonoid estimate variables (ICC: 0.73, 95% CI: 0.70–0.76) and excellent for categorical (quintile) flavonoid estimates (K: 0.89, 95% CI: 0.88-0.90). The Spearman's rank coefficient that examined the correlation was high for all comparisons (*r* > 0.9; Table 4).

**Table 4:** Degree of reliability and correlation in continuous and quintiles flavonoid intake estimations by databases and methods used.

**Comparison^a^**	**ICC (95% CI)**	**Kappa (95% CI)**	**Spearman's Rho**
PE - Total aglycones & PE - Total aglycones transformed.	0.99 (0.99–0.99)	0.98 (0.94–0.96)	0.99
PE - Total aglycones & PE - Total glycosides	0.97 (0.94–0.99)	0.96 (0.96–0.97)	0.98
PE - Aglycones tranformed & PE Total glycosides	0.98 (0.84–0.99)	0.96 (0.96–0.97)	0.99
PE - Total aglycones & USDA - Total aglycones	0.73 (0.70–0.76)	0.89 (0.88–0.90)	0.92
PE - Aglycones transformed. & USDA - Total aglycones	0.72 (0.68–0.76)	0.88 (0.87–0.90)	0.91
PE Total glycosides & USDA - Total aglycones	0.76 (0.73–0.77)	0.87 (0.86–0.89)	0.91

*PE, Phenol Explorer; USDA, United States Department of Agriculture; ICC, intraclass coefficient, worked as continuous variable; Kappa, Kappa weighted squared, worked as categorical variable (quintiles)*.

The degree of reliability and the correlation between methods for estimating flavonoid subclasses (i.e., flavanols, anthocyanins, flavanones, flavones, flavonols, and isoflavones) are presented in Supplementary Tables 3–8. When comparing the estimated intakes between the PE and USDA aglycone methods, the reliability was only excellent for categorical flavanol intakes and for both continuous and categorical flavanone intakes. For all subclasses, except flavanones and isoflavones, reliability estimates were better when intakes were modeled as a categorical variable. Importantly, the reliability between databases was poor for flavones (both continuous and categorical), flavonols (continuous), and isoflavones (categorical).

## Discussion

The main differences between the PE and USDA databases, such as the chemical structures, analytical methods, and the number of foods and phenolic compounds studied (6, 10, 34), are well known. However, these are not always taken into account when comparisons are made between studies using different methodologies. We report mostly high concordances among the 4 flavonoid intake estimation methods, especially when considering flavonoid intake as a categorical variable. The main comparison between total aglycones from PE and USDA was moderate reliability when the variable was continuous, which improved to excellent as a categorical variable by quintiles. This degree of reliability was similar for flavanols and flavanones, but lower for anthocyanins and flavanols and poorer for flavones and isoflavones. In this study, there was a relevant difference between databases on flavonoid intake at the 80^th^ percentile. When comparing the PE methods, the differences were consistent at all percentiles.

Comparing flavonoid intake in studies is complex, not only due to the inherent limitations of dietary assessment but also because of the type of dietary questionnaire, the FCDB used (including the version), and how the results are presented (i.e., mean or median). For example, several studies have reported that flavonoid intake is generally higher when the USDA databases, rather than the PE database, are used (12). A recent systematic review indicated that the majority of reported polyphenol estimates have been from FFQs, where the studies were designed for other purposes and where FFQs were mostly not validated for the polyphenol intake (5).

In the last decade, the number of polyphenol and flavonoid studies has increased, especially those using PE and USDA databases alone, in combination, or together with other databases (5). A few studies have estimated an average intake of flavonoids using both databases. For example, a Polish study found an average intake of 524.6 mg/day according to USDA and 403.5 mg/day according to PE (19), while an Australian study reported 834 mg/day using USDA and 487 mg/day using PE as the average intake of flavonoids (21), with the discrepancy between databases mainly attributed to the flavonoid estimation methods, the high tea consumption in this cohort, and the thearubigins inclusion. This is consistent with our findings that flavonoid intakes tend to be higher when estimated using USDA. However, two studies showed a different direction for total flavonoid intake estimation, likely because they considered flavonoids in their glycosylated form (22, 23). A Brazilian study presented a total flavonoid estimation of 86.6 mg/meal and 106 mg/meal in Food Service, using USDA and PE, respectively (22). Most recently, a US study of two large observational cohort studies showed that the total flavonoid mean intakes of men and women were higher in PE than in USDA (23). The discrepancies are mainly due to the food composition database (plus the version used) and whether the methodology included all kinds of phenolic compounds.

Only one study explored the association between flavonoid intake and risk of all-cause mortality using both databases, and similar results for total flavonoid consumption of 696 mg/d (median: 668) and 674 mg/d (median: 648) were presented (35). Although they used different databases (USDA vs. PE) and methods (glycosides vs aglycones) for the flavonoid estimation, they did not find differences in the final results. Various factors can influence the relation between health outcomes/all-cause mortality and flavonoid intakes such as population characteristics, flavonoid intake levels, and the design of the study, among others. Furthermore, in some epidemiological studies, the total intake of flavonoids and specific subclasses (i.e., flavone, isoflavone, and anthocyanin) have not been significantly associated with chronic diseases (12, 35). Some meta-analyses that associated dietary total flavonoid intake/subclasses of flavonoids and risk of mortality from all causes and cardiovascular disease did not consider differences in databases or methods used (36–39). Combining intakes estimated using glycosides and aglycones is not recommended, irrespective of the degree of reliability, since glycosides and aglycones may be differently associated with health outcomes than aglycones (40, 41). However, if the combination is necessary for the type of study, the recommendation is to use a similar analytical method (i.e., only aglycones). Thus, conclusions from studies using different assessment methods must be carefully interpreted.

To the best of our knowledge, only one study (21) has evaluated the correlation between intakes estimated using different databases and found a Pearson correlation coefficient of 0.94, which is very similar to Spearman's rank correlation coefficient of 0.92 in this study. The reliability of total flavonoid intake estimation is excellent among all the methods as a categorical variable (K: 0.87 to 0.98); however, it is moderated when it is continuous for both databases (ICC: 0.73–0.76). It is very important to mention the accuracy and the selection of databases, methods, and statistical tests because only using flavonoids database from USDA compared with total aglycones from PE database obtained a Spearman coefficient of 0.89 but a 0.43 ICC of agreement (Supplementary Table 1).

Overall, strong correlations were observed between methods for individual flavonoid subclasses, particularly for flavanols (0.92–0.99) and flavanones (0.80–0.99). These data are consistent with those reported by Ivey et al. where they showed a correlation of 0.89 for flavanol aglycones and 0.99 for flavanone aglycones between databases (21). Currently, limited data exist about the reliability and concordance of flavonoid subclasses. This is important as a high correlation does not mean that concordance is also high, as can be seen in the ICC of the flavanol subclass (Supplementary Table 3) comparing PE with USDA. Anthocyanidins reflect an essential case where the reliability between databases was only moderate; this could explain or contribute to different results in studies on chronic diseases (12, 38, 42). For isoflavones, heterogeneity of the results was observed for each comparison method, but intakes in this cohort were very low.

According to the subclass contributions presented in our study, it is necessary to emphasize that the discrepancies between the aglycone methods are extensive between anthocyanins, flavanols, flavones, and isoflavones, being more notable in these last two subclasses, both quantitatively and proportionally. In fact, other studies that use the PE and USDA databases showed similar trends according to flavonoid subclasses and food contribution data (21, 22). Our findings that USDA estimates were larger than the PE estimates for flavanols and anthocyanidins, while the PE estimates were greater than the USDA estimates for the flavonols and flavones are in agreement with the previously cited articles. Thus, the scientific references and the biocompounds that are included in the databases could influence these differences. For example, the low contribution of flavones from USDA could be related to the fact that they consider only two compounds, namely, apigenin and luteolin, whereas PE includes up to 15 (7). This could be explained by the chemical structure (C-glycoside) which is hard to hydrolyze, and therefore, the data in the USDA database are expected to be lower. Furthermore, the newly released updates could reduce the difference between the bases (6, 43). For example, the researchers found that anthocyanidins with USDA are about eight times greater than PE anthocyanidins; however, in our data set, this was reduced to 1.4 times (21).

### Food Sources

The sources of tea and cocoa products establish the most important differences between contributions of flavonoid intake by the database. The conflict between databases in estimating flavonoid intake from tea in this study is explained by the fact that USDA includes two scientific sources on thearubigins (6). Even though thearubigins could have a high impact on total flavonoid estimations, no proper analytical methods are available to quantify them (44). So, in the meantime, the current suggestion would be to not include thearubigins.

The differences observed between the databases were not related to the proportion of total flavonoid intakes or to the chemical structures. Cocoa products were higher in the contribution by 4.8% in aglycones PE than USDA on flavonoids; however, conflicts were observed in specific food sources. When analyzing dark chocolate, it can be seen that most of the proanthocyanidins contribute more in PE than USDA, reaching up to 55% of total proanthocyanidins. Also, PE considers flavonol compounds such as quercetin (21). Conversely, cocoa powder from USDA shows a higher contribution in proanthocyanidins, especially from polymers (> 10), up to 42.4% but decreasing to 24.3% when total proanthocyanidins are incorporated. The main explanation could be related to the scientific sources of both polyphenol databases (8, 9). Lastly, despite the differences observed and the proportions, the net estimation of cocoa products was 3.3% higher in aglycones USDA than PE.

As expected, the food sources related to the PE glycosides database reached a higher content of flavonoids than PE aglycones due to their chemical structure as glycosylated or esterified biocompounds include a higher molecular weight. This is independent of some lower proportions presented in Table 2. However, similar but lower contributions in fruits, nuts, and seeds were observed and explained by total proanthocyanidins. Mostly, it is possible to highlight a major contribution in vegetables, spices, and herbs by USDA and in cereals, nuts, and seeds by PE. Similar results about food contributions were found in the Polish study (19).

### Challenges and Recommendations

After comparing studies on flavonoids, we recommended using similar databases and methods for intake assessment applied; however, caution must be taken with the interpretation, specifically in some subclasses (i.e., flavonols, flavones, and isoflavones), as moderate to lower concordance was found. The selection of a database, method, and proper methodology will depend on the aim of the study, the population of interest, and their food consumption. The combining of databases/sources in an international system could be an interesting challenge to cover insufficient data for food items and missing values, among others. Indeed, international food databases need to move forward in updating foods and covering more flavonoid-rich foods from different regions (1, 22). Standardization of the procedures for the quantification of polyphenols is the key to a better validation and comparison of data. It is worth noting that the recommendations in this article are particularly focused on how flavonoid assessment methods are conducted/reported and on the approach described in the study. Furthermore, consideration should be given to the limitations and advancements in the field of flavonoids and their relationships with human health.

This study has two main strengths. The first is the statistical tests selected that were used for the reliability and correlation of the flavonoid data between methods. The second is the use of the most updated FCDB and several approaches regarding analytical methods and their chemical structure that have been used in the literature. However, this study has weaknesses related to the limitations of FCDBs in terms of incomplete data related to foods or polyphenols (analytical method or specific compounds) and also this study focuses only on flavonoids, avoiding the phenolic acid class, which are important contributors of total dietary polyphenol intakes (45). Moreover, it is important to bear in mind the potential measurement error of the self-reported 24-HDR, which usually underestimates the real intake of all foods, including those rich in polyphenols. However, this point will not excessively affect our comparisons between methods since we are using the same dietary questionnaire. Although we have compared four methods to estimate flavonoids, none of them could be considered as the gold standard, so it was not possible to point out which one was the most reliable.

## Conclusion

All comparison methods of estimated flavonoid intakes were similar, providing an excellent reliability between PE database pairs. When comparing PE and USDA total aglycones, there was a moderate reliability when a continuous variable was used, while the reliability was excellent when flavonoid intake was modeled as a categorical variable. The same trend was observed for flavanol, flavanone, and anthocyanidin subclasses with a high reliability, and a poor reliability for flavone, flavonol, and isoflavone subclasses. This difference is largely explained by the databases.

Researchers must be aware of the limitations of the databases and methods selected for flavonoids estimations and the consistency with the aim/design of the study. It is worth mentioning that the recommendation would be to use categorical variables if possible among the studied databases as better concordance can be obtained. It seems that this is more complex for some subclasses of flavonoids, and it requires analysis of the evidence of each class according to the method and statistical testing. Further research needs to examine more closely the reliability of flavonoids and their subclasses between methods or approaches.

## Data Availability Statement

The datasets presented in this article are not readily available due to the Master Agreements of the DiGuMet project requests to access the datas.

## Ethics Statement

The Diet, Cancer and Health - Next Generations research project was approved by the Danish Data Protection Agency (journal number 2013-41- 2043/2014-231-0094) and by the Committee on Health Research Ethics for the Capital Region of Denmark (journal number H-15001257). The participants provided their written informed consent to participate in the study.

## Author Contributions

FL, RZ-R, AT, RL, JH, and CA-L contributed to the conceptualization and methodology. AR-H, JH, and AT collected the data of the DCH-NG cohort. FL and AR-H computed the flavonoid dietary intake. FL performed the formal analysis and wrote the first draft of the manuscript. RZ-R, NB, and CA-L supervised the first draft of the manuscript. RL and CA-L obtained the funding for this analysis. AR-H, JH, NB, RL, and AT reviewed, edited, and contributed to the final version of the manuscript. All authors have read and agreed to the published version of the manuscript. All authors contributed to the article and approved the submitted version.

## Funding

This work was accomplished as part of the DiGuMet project Diet × gut microbiome-based metabotypes to determine cardio-metabolic risk and tailor intervention strategies for improved health supported within the European Joint Programming Initiative A Healthy Diet for a Healthy Life (http://www.healthydietforhealthylife.eu), granted by MINECO (Spain, PCIN-2017-076). The work also received funding from CIBERFES funded by the Instituto de Salud Carlos III and co-funded by the European Regional Development Fund's Away to make Europe and the Generalitat de Catalunya's Agency AGAUR (2017SGR1546). The DCH-NG cohort was supported by the Danish Cancer Society, Knæk Cancer 2012 and Den A.P. Møllerske Støttefond (grant no 10619). The establishment of the MAX-study was partly funded by FORMAS (DNR 2016-00314). FL was supported by the Chilean government for doing his PhD through the National Agency for Research and Development (ANID)/Food and Nutrition Doctoral Program/DOCTORADO BECAS CHILE/2019 – 72200061. RZ-R was supported by the Miguel Servet program (CPII20/00009) from the Institute of Health Carlos III [co-funded by the European Social Fund (ESF) – ESF investing in your future].

## Conflict of Interest

The authors declare that the research was conducted in the absence of any commercial or financial relationships that could be construed as a potential conflict of interest.

## Publisher's Note

All claims expressed in this article are solely those of the authors and do not necessarily represent those of their affiliated organizations, or those of the publisher, the editors and the reviewers. Any product that may be evaluated in this article, or claim that may be made by its manufacturer, is not guaranteed or endorsed by the publisher.
