# Supplementary material for: Comparison of Flavonoid Intake Assessment Methods Using USDA and Phenol Explorer Databases: Subcohort Diet, Cancer and Health-Next Generations—MAX Study
Source: Front Nutr. 2022 Apr 4;9:873774. doi: 10.3389/fnut.2022.873774 (PMC9014246; doi:10.3389/fnut.2022.873774)
Supplement: Supplementary file 1 [file Data_Sheet_1.docx]

Supplementary Material

**Supplementary Table 1:** Degree of reliability and correlation in continuous and quintile flavonoid intake estimations from flavonoids aglycones by USDA and other methods.

| **Comparison** | **ICC (95% CI)** | **Kappa (95% CI)** | **Spearman´s Rho** |
| --- | --- | --- | --- |
| USDA - Flavonoids &  PE - Total Aglycones | 0.43 (0.27-0.55) | 0.68 (0.65-0.70) | 0.89 |
| USDA - Flavonoids &  PE - Total Aglycones Transformed. | 0.43 (0.29-0.54) | 0.66 (0.63-0.69) | 0.88 |
| USDA - Flavonoids &  PE Total Glycosides | 0.45 (0.20-0.61) | 0.69 (0.66-0.72) | 0.87 |
| USDA - Flavonoids &  USDA - Total Aglycones | 0.47 (0.27-0.60) | 0.74 (0.72-0.77) | 0.75 |

PE: Phenol Explorer; USDA: United States Department of Agriculture. ICC: intraclass coefficient, worked as continuous variable; Kappa: Kappa weighted squared, worked as categorical variable (quintiles).

**Supplementary Table 2:** Examples of top food items by 100 g/ml from databases and methods used in MAX study.

| Top food items  (mg/100g) | Flavonoid Databases | | | |
| --- | --- | --- | --- | --- |
|  | Phenol-Explorer (PE) | | | USDA |
|  | Total aglycones | Total aglycones transformed^a^ | Total glycosides^b^ | Total aglycones |
| Cocoa products^c^ |  |  |  |  |
| Milk chocolate | 682 | 683 | 683 | 905 |
| Dark chocolate | 1193 | 1195 | 1195 | 1583 |
| Total fruits (raw) |  |  |  |  |
| Apple | 119 | 119 | 124 | 109 |
| Orange | 48 | 3.0 | 6.1 | 52 |
| Blueberry | 294 | 249 | 349 | 363 |
| Tea (mg/100ml) |  |  |  |  |
| Black tea | 66 | 68 | 83 | 122 |
| Green tea | 140 | 140 | 172 | 142 |
| Nuts and seeds |  |  |  |  |
| Almond (raw) | 183 | 183 | 184 | 164 |
| Bean^d^ | 318 | 318 | 331 | 272 |
| Wine (mg/100ml) |  |  |  |  |
| Red wine | 61 | 64 | 76 | 64 |
| Cereals and baked products |  |  |  |  |
| White bread boll/bun^e^ | 11 | 11 | 25 | 0 |
| Vegetables |  |  |  |  |
| Broccoli (boiled) | 14 | 14 | 28 | 2 |
| Spinach (raw) | 79 | 64 | 119 | 11 |

^a^Transformed (converted from glycosides by chromatography without hydrolysis); ^b^All forms (glycosides, aglycones and esters); ^c^milk chocolate (40-50% of cocoa) and dark chocolate (70-80% of cocoa).^d^Raw or canned kidney bean in USDA databases and others raw common bean in PE; ^e^considering wheat flour from PE. No data was available for USDA databases on flavonoids and isoflavones, while zero was the content for the USDA database on proanthocyanidins.

**Supplementary Table 3:** Degree of reliability and correlation in continuous and quintile **flavanol** intake estimations by databases and method.

| **Comparison** | **ICC (95% CI)** | **Kappa (95% CI)** | **Spearman´s Rho** |
| --- | --- | --- | --- |
| PE - Total Aglycones &  PE - Total Aglycones Transformed. | 0.99 (0.99-0.99) | 0.99 (0.99-0.99) | 0.99 |
| PE - Total Aglycones &  PE - Total Glycosides | 0.99 (0.99-0.99) | 0.99 (0.98-0.99) | 0.99 |
| PE - Total Aglycones Transformed. &  PE - Total Glycosides | 0.99 (0.99-0.99) | 0.99 (0.98-0.99) | 0.99 |
| PE - Total Aglycones &  USDA - Total Aglycones | 0.72 (0.67-0.75) | 0.90 (0.89-0.91) | 0.93 |
| PE - Total Aglycones Transformed. &  USDA - Total Aglycones | 0.72 (0.68-0.76) | 0.90 (0.89-0.91) | 0.93 |
| PE - Total Glycosides &  USDA - Total Aglycones | 0.75 (0.71-0.77) | 0.91 (0.90-0.93) | 0.94 |

PE: Phenol Explorer; USDA: United States Department of Agriculture. ICC: intraclass coefficient, worked as continuous variable; Kappa: Kappa weighted squared, worked as categorical variable (quintiles).

**Supplementary Table 4:** Degree of reliability and correlation in continuous and quintile **anthocyanidin** intake estimations by databases and its methods.

| **Comparison** | **ICC (95% CI)** | **Kappa (95% CI)** | **Spearman´s Rho** |
| --- | --- | --- | --- |
| PE - Total Aglycones &  PE - Total Aglycones Transformed. | 0.99 (0.99-0.99) | 0.99 (0.99-0.99) | 0.99 |
| PE - Total Aglycones &  PE - Total Glycosides | 0.84 (0.75-0.89) | 0.98 (0.98-0.99) | 0.99 |
| PE - Total Aglycones Transformed. &  PE - Total Glycosides | 0.84 (0.74-0.89) | 0.99 (0.98-0.99) | 0.99 |
| PE - Total Aglycones &  USDA - Total Aglycones | 0.68 (0.64-0.72) | 0.76 (0.73-0.80) | 0.80 |
| PE - Total Aglycones Transformed. &  USDA - Total Aglycones | 0.67 (0.63-0.71) | 0.76 (0.73-0.79) | 0.80 |
| PE - Total Glycosides &  USDA - Total Aglycones | 0.64 (0.60-0.67) | 0.76 (0.73-0.79) | 0.80 |

PE: Phenol Explorer; USDA: United States Department of Agriculture. ICC: intraclass coefficient, worked as continuous variable; Kappa: Kappa weighted squared, worked as categorical variable (quintiles).

**Supplementary Table 5:** Degree of reliability and correlation in continuous and quintile **flavanone** intake estimations by databases and methods.

| **Comparison** | **ICC (95% CI)** | **Kappa (95% CI)** | **Spearman´s Rho** |
| --- | --- | --- | --- |
| PE - Total Aglycones &  PE - Total Aglycones Transformed. | 0.35 (0.28-0.42) | 0.86 (0.85-0.88) | 0.90 |
| PE - Total Aglycones &  PE - Total Glycosides | 0.51 (0.47-0.55) | 0.87 (0.85-0.88) | 0.91 |
| PE - Total Aglycones Transformed. &  PE - Total Glycosides | 0.76 (0.68-0.82) | 0.97 (0.97-0.98) | 0.99 |
| PE - Total Aglycones &  USDA - Total Aglycones | 0.97 (0.96-0.97) | 0.83 (0.81-0.86) | 0.87 |
| PE - Total Aglycones Transformed. &  USDA - Total Aglycones | 0.33 (0.68-0.76) | 0.76 (0.73-0.79) | 0.80 |
| PE - Total Glycosides &  USDA - Total Aglycones | 0.45 (0.41-0.49) | 0.79 (0.76-0.82) | 0.83 |

PE: Phenol Explorer; USDA: United States Department of Agriculture. ICC: intraclass coefficient, worked as continuous variable; Kappa: Kappa weighted squared, worked as categorical variable (quintiles).

**Supplementary Table 6:** Degree of reliability and correlation in continuous and quintile **flavone** intake estimations by databases and methods.

| **Comparison** | **ICC (95% CI)** | **Kappa (95% CI)** | **Spearman´s Rho** |
| --- | --- | --- | --- |
| PE - Total Aglycones &  PE - Total Aglycones Transformed. | 0.92 (0.88-0.94) | 0.87 (0.85-0.89) | 0.90 |
| PE - Total Aglycones &  PE - Total Glycosides | 0.71 (0.52-0.81) | 0.87 (0.85-0.89) | 0.90 |
| PE - Total Aglycones Transformed. &  PE - Total Glycosides | 0.70 (0.37-0.83) | 0.98 (0.98-0.99) | 0.99 |
| PE - Total Aglycones &  USDA - Total Aglycones | 0.09 (0.01-0.16) | 0.23 (0.18-0.28) | 0.26 |
| PE - Total Aglycones Transformed. &  USDA - Total Aglycones | -0.01 (-0.05-0.03) | 0.02 (-0.02-0.07) | 0.02 |
| PE - Total Glycosides &  USDA - Total Aglycones | -0.006 (-0.04-0.03) | 0.01 (-0.03-0.06) | 0.01 |

PE: Phenol Explorer; USDA: United States Department of Agriculture. ICC: intraclass coefficient, worked as continuous variable; Kappa: Kappa weighted squared, worked as categorical variable (quintiles).

**Supplementary Table 7:** Degree of reliability and correlation in continuous and quintile **flavonol** intake estimations by databases and methods.

| **Comparison** | **ICC (95% CI)** | **Kappa (95% CI)** | **Spearman´s Rho** |
| --- | --- | --- | --- |
| PE - Total Aglycones &  PE - Total Aglycones Transformed. | 0.97 (0.97-0.98) | 0.92 (0.91-0.94) | 0.95 |
| PE - Total Aglycones &  PE - Total Glycosides | 0.78 (0.62-0.86) | 0.92 (0.91-0.94) | 0.95 |
| PE - Total Aglycones Transformed. &  PE - Total Glycosides | 0.79 (0.59-0.87) | 0.97 (0.97-0.98) | 0.99 |
| PE - Total Aglycones &  USDA - Total Aglycones | 0.35 (0.31-0.40) | 0.66 (0.62-0.69) | 0.69 |
| PE - Total Aglycones Transformed. &  USDA - Total Aglycones | 0.31 (0.26-0.36) | 0.63 (0.60-0.67) | 0.66 |
| PE - Total Glycosides &  USDA - Total Aglycones | 0.18 (0.12-0.25) | 0.63 (0.60-0.67) | 0.67 |

PE: Phenol Explorer; USDA: United States Department of Agriculture. ICC: intraclass coefficient, worked as continuous variable; Kappa: Kappa weighted squared, worked as categorical variable (quintiles).

**Supplementary Table 8:** Degree of reliability and correlation in continuous and quintile **isoflavone** intake estimations by databases and methods.

| **Comparison** | **ICC (95% CI)** | **Kappa (95% CI)** | **Spearman´s Rho** |
| --- | --- | --- | --- |
| PE - Total Aglycones &  PE - Total Aglycones Transformed. | 0.99 (0.99-0.99) | 0.82 (0.80-0.85) | 0.92 |
| PE - Total Aglycones &  PE - Total Glycosides | 0.88 (0.85-0.90) | 0.82 (0.80-0.85) | 0.92 |
| PE - Total Aglycones Transformed. &  PE - Total Glycosides | 0.86 (0.84-0.89) | 0.99 (0.99-0.99) | 0.99 |
| PE - Total Aglycones &  USDA - Total Aglycones | 0.58 (0.53-0.62) | 0.26 (0.20-0.31) | 0.31 |
| PE - Total Aglycones Transformed. &  USDA - Total Aglycones | 0.49 (0.43-0.55) | 0.31 (0.24-0.37) | 0.43 |
| PE - Total Glycosides &  USDA - Total Aglycones | 0.65 (0.60-0.69) | 0.31 (0.24-0.37) | 0.43 |

PE: Phenol Explorer; USDA: United States Department of Agriculture. ICC: intraclass coefficient, worked as continuous variable; Kappa: Kappa weighted squared, worked as categorical variable (quintiles).
